# Using the theoretical domains framework to inform strategies to support dietitians undertaking body composition assessments in routine clinical care

**DOI:** 10.1186/s12913-021-06375-7

**Published:** 2021-05-28

**Authors:** Chloe J. D. Jobber, Shelley A. Wilkinson, Elyssa K. Hughes, Fiona Nave, Barbara S. van der Meij

**Affiliations:** 1 Department of Dietetics & Foodservices, Mater Health, South Brisbane, Queensland Australia; 2 School of Human Movement and Nutrition Sciences, The University of Queensland, Brisbane, Queensland 4072 Australia; 3 Nutrition & Dietetics, Mater Research Institute - The University of Queensland, Brisbane, Queensland Australia; 4 Department of Nutrition and Dietetics, Logan Hospital, Meadowbrook, Queensland Australia; 5 Bond University Nutrition and Dietetics Research Group, Gold Coast, Queensland Australia

**Keywords:** Barriers, Body composition assessment, Enablers, Malnutrition, Sarcopenia

## Abstract

**Background:**

Malnutrition, sarcopenia and cachexia are clinical wasting syndromes characterised by muscle loss. Systematic monitoring by body composition assessment (BCA) is recommended for the diagnosis, treatment and monitoring of the syndrome(s). This study investigated practices, competency, and attitudes of Australian dietitians regarding BCA, to inform a local implementation process.

**Methods:**

Applying the Action cycle in the Knowledge to Action framework, surveys were distributed to the 26 dietitians of an 800-bed tertiary hospital. The survey assessed barriers and enablers to performing routine BCA in clinical care. Results were categorised using the Theoretical Domains Framework (TDF) and suitable interventions mapped using the Behaviour Change Wheel.

**Results:**

Twenty-two dietitians (84.6%) completed the survey. Barriers to BCA were identified in all TDF domains, particularly in Knowledge, Skills, Social/professional role and identity, Beliefs about capabilities, and Environmental context and resources. Enablers existed in domains of: Skills; Beliefs about consequences; Goals; Environmental context and resources; Social influences; Intentions; Optimism; Reinforcement.

**Conclusions:**

This study showed that hospital dietitians experience individual, team, and organisational barriers to adopt BCAs in clinical practice. We were able to formulate targeted implementation strategies to overcome these barriers to assist BCA adoption into routine practice.

**Supplementary Information:**

The online version contains supplementary material available at 10.1186/s12913-021-06375-7.

## Background

Malnutrition, sarcopenia and cachexia are clinical wasting syndromes, prevalent in patients with acute or chronic diseases and frail elderly [1–3]. Malnutrition occurs in 20–50% [1, 4] of patients in acute care settings, sarcopenia in 15–70% [5, 6] and cachexia in 5–80% [7–9]. Malnutrition is defined as “*a state resulting from lack of intake or uptake of nutrition that leads to altered body composition (decreased fat free mass) and body cell mass leading to diminished physical and mental function and impaired clinical outcome from disease*” [10]. Malnutrition is associated with reduced treatment efficacy and increased healthcare costs [11, 12]. Disease-related malnutrition is characterised by inflammation and can be acute or chronic. Chronic disease-related malnutrition is also called ‘cachexia’ and is characterised by *“inflammation and ongoing loss of weight and muscle mass”* [10]. Sarcopenia is a condition which is often associated with malnutrition. It is defined as “*loss of skeletal muscle mass and strength related to ageing and/or chronic disease*” [13, 14], and is associated with negative outcomes across health care settings including reduced survival, worse clinical outcomes and impaired quality of life in many clinical populations including oncology, surgical, hepatology, and older adults [15–17]. As sarcopenia is prevalent amongst elderly and chronically ill, assessment and treatment has been encouraged by several leading expert groups [13, 18]. To be able to identify sarcopenia, assessment of muscle strength and muscle quantity or quality is required.

The three syndromes of malnutrition, cachexia, and sarcopenia are present in hospital populations and although they have been well defined in clinical practice, the umbrella term ‘malnutrition’ is used for patients who show signs of inadequate food intake, weight loss, and muscle wasting. It is recommended to screen for malnutrition on admission to the hospital and regularly during hospital stay, and to treat malnutrition as early as possible [10, 19, 20]. Malnutrition is typically ‘managed’ with a two-step process of screening and assessment. The initial step uses a malnutrition screening tool, such as the Malnutrition Screening Tool (MST) or Nutritional Risk Screening (NRS) [10, 21]. Patients classified as ‘at risk of malnutrition’ are subsequently referred to a dietitian. The second step is a dietitian assessment using a validated tool, such as the Subjective Global Assessment (SGA) or Mini-Nutritional Assessment (MNA) [10, 11]. These assessment tools diagnose malnutrition by drawing on objective parameters such as weight and metabolic demand, as well as subjective parameters like weight history, nutrition impact symptoms, and physical examination of muscle mass and subcutaneous fat stores [22]. Nutritional assessment may include additional anthropometric assessments such as mid upper arm circumference, skin fold thickness and mid upper arm muscle circumference [23].

Whilst parameters of nutrition assessment tools are easy to gather and rate highly regarding sensitivity, specificity and inter-rater reliability, they do not provide objective data on body composition such as muscle mass [24]. In addition, there is a subset of patients who cannot be weighed and using an estimated weight leaves a margin for error in classifying malnutrition [25]. Nutrition assessment tools also fail to recognise that patients can have a low level of lean tissue with any BMI category [26].

Thus, measures of overall weight loss lack the sensitivity to detect the amount of lean mass an individual has and the potential loss of lean mass experienced. This introduces the potential to grossly underestimate the prevalence of hospital malnutrition if diagnosis is based on body weight and body weight changes alone. Other challenges that impact the correct identification of malnutrition are our ageing society and the global epidemic of overweight and obesity [27], resulting in a higher number of patients with sarcopenia, as well as overweight and obese patients with chronic or acute diseases [28–30]. Malnutrition in these patients is harder to recognise using the aforementioned assessment tools, but is nonetheless associated with worse outcomes [25, 31].

As a result of these shortcomings there is a lack of consensus amongst the dietetic and medical community surrounding malnutrition assessment methodologies [17, 32–37]. With societal changes and improved technologies available, it is prudent to consider additional objective ways to obtain information on lean mass, in order to diagnose and monitor the effectiveness of the treatment of malnutrition [17, 37, 38].

Routinely measuring lean mass provides an objective measure to diagnose and monitor malnutrition. This aligns with the international clinical nutrition community’s recognition of the need for BCA as part of malnutrition assessment [15, 17, 39]. Indeed, the new Global Leadership Initiative on Malnutrition (GLIM) criteria recommends the measuring of body composition and identifying loss of lean mass as one of the top five criteria to assist in diagnosing malnutrition [27, 33]. Since the launch of the GLIM criteria, several initiatives have been taken to validate the criteria. These showed that the GLIM criteria have a fair agreement with the reference standard [40–42].

Despite available evidence of the benefits of BCA, body composition is not routinely used by dietitians in clinical practice. This is reportedly due to incomplete knowledge and awareness, uncertainty of how and when to measure, poor availability of assessment tools and a lack of time [17, 43]. Given the role that lean mass plays in the clinical outcomes of certain illnesses, it is of critical importance that its assessment be added into the nutrition field [15, 17].

The most common BCA techniques that have been validated for use in humans are skinfold measurements, single and multi-frequency bio-impedance analysis, hydrodensitometry, Dual Energy X-ray Absorptiometry (DXA), computerized tomography (CT)-scans, and air displacement plethysmography (E.g. BodPod) [44]. These techniques are all non-invasive but vary with regards to cost, precision and validity, with skinfold measurements and bio-impedance analysis being relatively imprecise and DXA, CT-scans and air displacement plethysmography being more accurate but also more costly techniques, and less appropriate for bedside measurements [15, 44, 45]. Recently, ultrasound techniques have been used to assess body compartments at the bedside, for instance the upper quadriceps muscle [15].

It is widely recognised that the dissemination of information alone does not change practice [46]; thus drawing on an implementation science methodology facilitating this change and adoption process [47]. This theory-driven approach guides the rigorous and systematic processes of evidence selection, adapting knowledge to the local context, understanding barriers and enablers to its use, selecting appropriate interventions to support its adoption, and monitoring and evaluating outcomes, as well as sustaining knowledge use, as outlined in the Knowledge-to-Action (KTA) framework [48]. Within this framework additional theories, models, and frameworks can be applied to guide structured and systematic barrier identification and intervention selection, such as the Theoretical Domains Framework (TDF) and the Behaviour Change Wheel (BCW) [49, 50].

The aim of this project was to develop a department-wide strategy to incorporate BCA by dietitians into routine clinical care in an 800-bed tertiary hospital in South-East Queensland (Australia) using an Implementation Science approach. To inform this process we planned to investigate the current local practices, competency, and attitudes of our departmental clinical dietitians with regards to the utilisation of BCA.

## Methods

The study was declared as Exempt from Review – Not Research according to the Human Research Ethics Committee of Mater Research Institute – UQ Human Research Ethics Committee (Project ID: EXMT/MML/58778). All methods were carried out in accordance with relevant guidelines and regulations. Informed consent was obtained from all participants; instructions for survey completion indicated their completion implied consent.

This implementation planning project occurred in an 800-bed tertiary hospital in Brisbane, Australia. The hospital provides services to both private and public inpatients and outpatients and includes a variety of patient populations. At the start of this project (May 2017), The Dietetics and Foodservices department consisted of 20.55 full time equivalents (FTE) with 26 dietitians.

To develop our BCA implementation strategy we followed the KTA framework which is an iterative approach that allows building (Knowledge Creation) and application of knowledge (Action Cycle) [40, 48, 51]. The Action Cycle was the focus of this work; with steps that can occur sequentially or concurrently and involve identification of the problem, assessing knowledge use determinants, evaluating the impact of knowledge use or outcomes, and ensuring sustainability [40]. The KTA is a ‘process model’ that guides the process of translating research into practice [48]. The KTA is flexible enough to enable some of the steps (e.g. ‘assess barriers to knowledge use’ and ‘select, tailor, implement interventions’) to be guided by further application of ‘Determination Theories’ (i.e TDF) [50] and ‘Implementation Theories (Theories’ (i.e. BCW) [49] to assess barriers and enablers and design suitable interventions [51]. The TDF is used as a system for categorising and defining barriers, and the BCW as a system for guiding decision-making around designing behaviour change interventions based on the identified barriers.

Below, we outline the survey process which allowed determination of a dietetic departmental practices, competency, and attitudes. A survey was developed to assess barriers and enablers to BCA use within the dietetic department (Additional file 1). Questions were designed by the authors to map against domains of the TDF [50]. Questions covered knowledge attitudes on, and confidence in BCA device use, frequency and predicted time taken to use the devices, views on how it would change dietetic practice, and which patient cohorts would benefit from BCA, informed by current literature on barriers and enablers to undertaking BCA [43] and discussion within the research team. Each question also had an ‘other’ option. All department dietitians were invited to complete the survey via an email link to an online survey portal (Survey Monkey, San Mateo, CA, USA) in May 2018. The survey was open for two weeks and two reminders were sent prior to the closing date.

The results were summarised as frequencies and percentages of answers for each question. All authors reviewed the summarised survey results and tabulated the barriers and enablers identified (Table 1). This process involved an analysis using the TDF as the framework to categorise responses into domains; these responses were sorted into identified barriers and enablers, followed by documenting the source of the behaviour using the BCW (column 5), potential behaviour change techniques (BCT) in column 6, and finally, interventions designed drawing from the implementation science literature (column 7) [49, 52]. Definitions of the BCW intervention components (column 5) are as follows: Education (Increasing knowledge or understanding); Persuasion (Using communication to induce positive or negative feelings or stimulate action); Incentivisation (Creating expectation of reward); Coercion (Creating expectation of punishment or cost); Training (Imparting skills); Restriction (Using rules to reduce the opportunity to engage in the target behaviour (or to increase the target behaviour by reducing the opportunity to engage in competing behaviours)); Environmental restructuring (Changing the physical or social context); Modelling (Providing an example for people to aspire to or imitate); and Enablement (Increasing means/reducing barriers to increase capability or opportunity) [49]. Findings were refined through group discussion resulting in consensus, with subsequent operationalisation and prioritisation of strategies, listed in column 7, informed by BCTs in column 6. The group consisted of two clinician-researchers (one with expertise in implementation science and one in body composition) who were embedded in the department and three clinicians (including one senior team leader) with an interest in body composition assessment and who also had a strong clinical understanding of the department.

**Table 1:** Intervention mapping and operationalising after sorting of barriers and enablers to TDF domains from the dietitians surveyed

TDF domain	n survey question respondents (from 22 dietitians)	Survey identified Barriers (% who reported barrier)	Survey identified Enablers (% who reported enablers)	BCW Intervention components and intervention definition	Behaviour change techniques (BCTs)	Potential strategies, operationalised as:
**Knowledge**	16	Unsure of clinical areas BCA would benefit 54.5% unsure who to use on 50.0% unsure when to do 50.0% unsure what to do 45.5% unsure how to interpret		***Psychological capability*** Education Training Enablement	Increasing knowledge or understanding: E.g. *Feedback on the behaviour/ outcome(s) of the behaviour* *Self-monitoring of behaviour/ of outcome of behaviour* *Prompts/cue* *Information about social and environmental consequences* *Information about others’ approval* *Imparting skills* Reducing barriers to increase capability or opportunity (beyond education, training and environmental restructuring) E.g. *Social support* *Reduce negative emotions* *Conserve mental resources* *Self-monitoring of behaviour and outcome of behaviour* *Graded tasks* *Adding objects to the environment* *Restructuring the social environment* *Focus on past success* *Verbal persuasion about capability* *Self-reward* *Goal setting (behaviour, outcome)* *Commitment* *Action planning* *Review behaviour and outcome goal(s)* *Discrepancy between current behaviour and goal* *Problem solving* *Pros and cons* *Monitoring of emotional consequences* *Anticipated regret*	• PD sessions (KPI: ≥ 3/y, ≥ 15 attendees) (Topics: Body Composition Assessment - overview; Practical on how to assess and interpret BCA; Case Study – diagnosis and follow up; Implementation plan; Sarcopenia) • Workshops to practice all BCA procedures (KPI:≥ 2/y, ≥ 2 attendees) • BPI and WAR updating exercise by dietitians: literature review and integrating evidence and procedures into BPIs and WARs • Information sharing from WARs and BPIs amongst teams in the department • Clinical champions – 6 month graded WAR adoption project using accountability, peer modelling and influence. • Set goals on increasing numbers of BCAs in eligible patients in each area • Feedback in department meeting after 3 months, new goal setting • Use social support: peer support within streams, clinical champions assisting and upskilling peers, reporting in streams and department meetings, BC team members meeting with individual staff members and helping to get body comp Ax running. • Discussion and sufficient preparation and support to decrease negative emotions. • Problem solving: provide resources (lanyards, literature, information folder) • Monthly meetings including mentoring to allow reflection on wins; set personal goals and rewards; action planning (also provided by 6 month project plan with action planning)
**Skills**	16	27.3% Don’t know how to use 18.2% Lack of confidence (and enabler) 18.2% Don’t have time to perform Never use: skinfold 90.9%, BIS 81.8%, handgrip 68.2%, MUAC 68.2% 54.5% unsure who to use on 50.0% unsure when to do 50.0% unsure what to do 45.5% unsure how to interpret	Training and awareness in a variety of areas Had any training in BCA: 54.5%, mostly in MUAC (40.9%); skinfolds (40.9%%), BIS (22.7%); DXA (4.5%) The majority of the team are aware that skinfold callipers (68.2%), BIS device (77.3%), handgrip dynamometer (68.2%), and tape measures (68.2%) are available. A smaller number is aware of the existence of a BIS scale (40.9%) Confident to use skinfold (4.5%), BIS (27.3%), MUAC (63.6%), PG-SGA (77.3%), handgrip (45.5%, tape measure (68.2%)	***Reflective motivation*** (**Cognitive/ interpersonal skills)** Education Persuasion and/or Incentivisation and/or Coercion ***Physical capability (physical skills)*** Training Enablement	Increasing knowledge or understanding E.g. *as above* Using communication to induce positive or negative feelings or stimulate action E.g. *Feedback on the behaviour/ on the outcome(s) of the behaviour,* *Focus on past success,* *Verbal persuasion about capability,* *Persuasive source* *Identity associated with changed behaviour* *Identification of self as role model,* *Information about social and environmental consequences,* *Information about health consequences,* *Salience of consequences,* *Information about others’ * *Social comparison* Creating expectation of reward E.g. *Feedback on behaviour or* *on the outcome(s) of behaviour,* *Self-monitoring of behaviour or outcome of behaviour,* *Monitoring of (outcome of) behaviour by others without evidence of feedback,* *Situation-specify reward,* *Reward incompatible behaviour,* *Reduce reward frequency,* *Reward alternate behaviour,* *Remove punishment,* *Social reward,* *Self-reward,* *Behavioural contract,* *Commitment,* *Discrepancy between current behaviour and goal* Creating expectation of punishment or cost As above	As above; incorporate some of the information (in PD or in mentoring) to tap persuasion: • ‘past’ successes (report on project process – either champion or BC team), • Goal setting and verbal persuasion about capability in mentoring Incorporated into project plan and engagement and reporting strategy for and with clinical champions (6 month project) As above, esp. technical skill development
**Social/ professional role and identity**	6	18.2% I think these measures are more appropriate for research 18.2% I do not think these measurements are appropriate for my area of work		***Reflective motivation*** Education Persuasion Incentivisation Coercion	As ‘Skills’	As above
**Beliefs about capabilities**	12	27.3% I don’t think I could perform these measures accurately 18.2% I do not have time to perform these measurements		***Reflective motivation*** Education Persuasion Incentivisation Coercion	As ‘Skills’	As above, especially how to be accurate As above, especially workflow practices (decide and discuss as a team/s)
**Beliefs about consequences**	5 (barriers) 19 (enablers)	13.6% Don’t think these measurements would benefit my practice/tell me anything new/useful 9.0% I do not expect these measurements to change my practice	77.3% Ability to more accurately assess energy requirements 72.3% Ability to provide objective measures/ evaluations of dietetic interventions 68.2% Assist in motivation (i.e. to continue on weight loss journey) 63.6% Would make practice more interesting 54.5% Assist in persuading patients to increase intake/supplements 50.0% Assist in identifying malnutrition 45.5% Would improve my practice 22.7% Leverage for nasogastric tubes 22.7% Leverage for pre-surgical provision of enteral/parenteral nutrition 4.5%With training and time BCAs could become routine	***Reflective motivation*** Education Persuasion Incentivisation Coercion	As ‘Skills’	As above, especially reflected in the BPIs and WARs – how this may be clinically relevant to measure and monitor; how to make routine; how to monitor; also areas for future research
**Goals**	16	54.5% unsure who to use on 50.0% unsure when to do 50.0% unsure what to do 45.5% unsure how to interpret	72.2% I would like to learn more about BCA 68.2% I would like to apply measurement of body composition to my practice 4.5%Make results more meaningful in practice 4.5% Applicable in some patient groups	***Reflective motivation*** Education Persuasion Incentivisation Coercion	As ‘Skills’	As above
**Memory, attention and decision processes**	16	59.1% Not in my daily routine 40.9% Hassle to find reference ranges 31.8% Too much time to do 22.7% I forget about doing or scheduling a measurement 4.5% Difficulties – practicalities	4.5% Great that we will have support to routinize	***Psychological capability*** Education Training Enablement	As ‘Knowledge’	As above, especially eventually formalise a process of documenting, trialling, evaluation in each WAR; also to consider new staff orientation
**Environmental context and resources**	10 15	31.8% We do not have procedures or forms to report these measurements 54.5% I don’t know how to book these devices 27.3% I don’t know where these devices are kept 18.2% I know where these devices are kept but I don’t know how to get them to the ward 18.2% I don’t have access to the devices I need to perform body composition assessment	4.5% If you can get access to the peapod for routine assessments that would be great	***Physical opportunity*** Restrictions Environmental restructuring	Using rules to reduce the opportunity to engage in the target behaviour (or to increase the target behaviour by reducing the opportunity to engage in competing behaviours) Changing the physical or social context	As above As above, plus purchase of new equipment; process of storing; booking; transporting; cleaning; lanyard ready reckoners
**Social influences**	13	18.2% My peers do not perform these measurements, so why should I? 4.5% I think they are burdensome to patients	4.5% I feel this would add value to Dietitians and patient care in relevant populations	***Social opportunity*** Restrictions Persuasion	As above	As above
**Intentions**	18	59.1% Not in my daily routine 31.8% I never think of doing these measurements when I see or evaluate a patient	4.5%I would like to know more about what technology we have available and where it would be applicable. 4.5%I would certainly consider integrating into practice if and where appropriate. 4.5% I would like to add these measurements to my daily routine	***Reflective motivation*** Education Persuasion Incentivisation Coercion	As ‘Skills’	As above
**Emotion**	14	27.3% Feel stressed about the time required 9.1% Don’t want to break device	4.5% Keen to get started	***Automatic motivation*** Persuasion Incentivisation Coercion Environmental restructuring Modelling Enablement	As above	‘Operationalise and integrate’ into BAU; 6/12 clinical champions project; reported back at teams (EBP & Research Dept meeting) – standing agenda item; future reporting ideas - Audit and feedback (w/ outcomes/positive wins to be shared) Formalised as “Best BCA adopter” – acknowledged at end of year
**Optimism**	19	4.5% Unsure if it’ll be burdensome to patients 4.5% Unsure how receptive the patients will be	63.6% Will make practice more interesting 45.5% Would improve my practice 4.5% May increase patients’ motivation to see me to get results 4.5% I am ready – bring it on	***Reflective motivation*** Education Persuasion Incentivisation Coercion	As ‘Skills’	As above, especially ensure Monitor and reflect upon benefits (e.g. for next 6 months as extra KPI for reflection – actual measures or ease of measuring outcomes and patient process)
**Reinforcement**	18	18.2% Nothing that prompts me	63.6% BCA team makes this possible 54.5% More training would prompt me 4.5% Integrate into WARs	***Automatic motivation*** Persuasion Incentivisation Coercion Environmental restructuring Modelling Enablement	As above	Ensure prompts are incorporated into standard procedures and documents (i.e. WARs) at end of 6/12 BCA clinical champion project
**Behavioural regulation**	18	45.5% I would need to change my practice regarding assessing nutritional status 31.8% Would need to change practice 18.2% Happy with the way I assess nutritional status	4.5% Happy to practice if measurements will improve patient care	***Psychological capability*** Education Training Enablement	As ‘Knowledge’	BPIs and WARS; especially focussing on Ax of nutritional status Incorporate into standard processes and procedures – explore and refine with Dept in subsequent PDs and Dept meetings in a planned way

*BPI* Best Practice Investigation; *WAR* Work Area Resource; *Dept* Department; *PD* professional development; *BCA* Body Composition Analysis; *TDF* Theoretical Domains Framework, *Ax* assessment, *DXA* Dual X-ray Absorptiometry

## Results

**Fig. 1:**
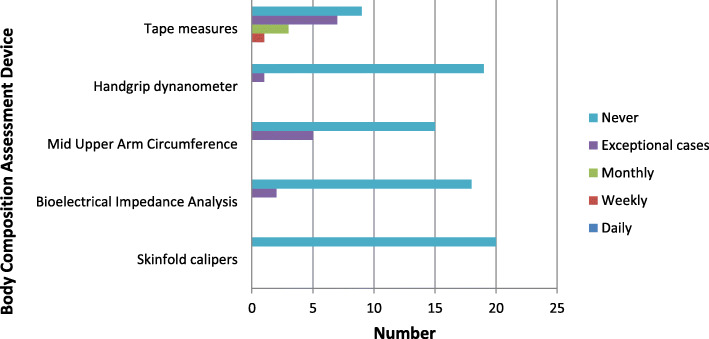
Frequency of reported device use by dietitians in routine clinical care

Twenty-two of 26 dietitians (84.6%) completed the survey. As shown in Table 2, more than half of clinicians had previous training in BCA, mostly in skinfold thickness and mid upper arm circumference (MUAC). Few had training in bioelectrical impedance spectroscopy (BIS) devices. The majority of clinicians were aware that skinfold calliper, BIS, PG-SGA physical exam, hand grip dynamometer and tape measure devices were available for use in their department. More clinicians felt confident using PG-SGA physical exam and tape measures with fewer feeling confident using the BIS, MUAC and handgrip devices and techniques. As seen in Fig. 1, the PG-SGA physical exam was the most common assessment reported to be performed, followed by the use of tape measures. The majority of clinicians reported that they never used skinfold measurement, BIS, MUAC or handgrip measures.

**Table 2:** Dietitian’s prior training, awareness of available devices and confidence in performing body composition assessments

% (n)	Dietitians
Response rate	84.6 (22)
Previous training in BCA use	
Yes	54.5 (12)
No	45.5 (10)
Previous training in BCA devices	
Skinfold callipers	40.9 (9)
Mid-upper arm circumference (MUAC)	40.9 (9)
Bioelectrical impedance spectroscopy (BIS)	22.7 (5)
Dual x-ray absorptiometry (DXA)	4.5 (1)
Knowledge of available of devices and procedures in department	
PG-SGA physical exam	86.4 (19)
BIS	81.8 (18)
Tape measures	77.3 (17)
Skinfold callipers	68.2 (15)
Handgrip dynamometer	68.2 (15)
Bioelectrical impedance scale	40.9 (9)
Rating of confidence in using BCA devices or undertaking procedures (extremely / reasonably confident)	
PG-SGA physical exam	77.3 (17)
Tape measures	68.2 (15)
MUAC	63.6 (14)
Handgrip dynamometer	45.5 (10)
BIS	27.3 (6)
Skinfold callipers	4.5 (1)

*BCA* body composition assessment; *BIS* Bioelectrical impedance spectroscopy; *DXA* dual x-ray absorptiometry; *MUAC* mid upper arm circumference; *PG-SGA* patient generated subjective global assessment; *REE* resting energy expenditure

Dietitians’ attitudes to use of BCA in routine practice were categorised barriers and enablers across TDF domains (Table 1). Around half of the dietitians reported not being sure who (54.5%), when (50%), what to do (50%), or how to interpret (45.5%) BCAs (TDF domain -Knowledge). Further, between 68.2–100% of BCA techniques were not used in their practice (Skills). Broadly, in their daily practice, the dietitians noted that undertaking BCAs were not in their daily routine (Belief about Capabilities; Memory, attention and decision processes; Intentions). Almost half (45.5%) felt they would need to change their practice to incorporate BCA into their assessments (Memory, attention and decision processes) and 40.9% reported it would be a hassle to find references ranges (Behavioural Regulation).

However, positively, over two-thirds of dietitians were aware of most of the devices in the department (Skills), and felt adding BCA into their practice would have a positive effect on a range of activities, including assessment of energy requirements (77.3%), providing objective measures of their interventions (72.3%) (Beliefs about Consequences). A large majority of dietitians reported they would like to learn more about BCAs (72.2%) and apply them in their practice (68.2%)(Goals), feeling it would make their practice more interesting (Optimism). The dietitians also agreed that the Body Composition team would make these changes possible (67.7%)(Reinforcement).

Table 1 shows the mapping of the identified barriers and enablers (columns 2 and 3) to the TDF domains (column 1). Interventions and how these can be operationalised, drawing from the literature [49, 52] are in columns 4 and 5. Barriers to use of BCA within our department were identified in all TDF domains. Enablers included: Skills; Beliefs about consequences; Goals; Environmental context and resources; Social influences; Intentions; Optimism; Reinforcement.

Through the detailed mapping process, these are summarised in and operationalised in column 6 of Table 1. They can broadly be grouped as: 1. Professional development strategy, 2. Body composition assessment clinical champion project, and 3. Departmental integration process.

## Discussion

This study aimed to understand the attitudes, beliefs, and practices of clinicians in a tertiary hospital dietetics department regarding patients’ BCA practices to inform a process of integrating these practices into routine clinical care. Most dietitians rarely used BCA with their patients in a systematic way. Barriers and enablers existed in many of the same TDF domains. Many dietitians felt unsure of their skills, when and how to systematically use these BCA techniques, and some questioned their benefit for particular clinical areas (E.g. neonatal care) and/or outside of research projects. However, many dietitians were optimistic about the potential this process would provide to enabling evidence-based practice and noted it would add to the strength of assessments, recommendations, and ability to detect malnutrition and other wasting syndromes, and to clinically relevant improvements within the delivery of medical nutrition therapy.

To our knowledge, this is the first study to investigate barriers and enablers to systematic adoption of BCA techniques into routine dietetic clinical practice. While many papers have promoted the use of BCA to detect malnutrition [10, 15, 17, 33, 36, 37], and specific studies described the application of these techniques in clinical areas (e.g. elderly [38]; liver failure [53–55]; oncology [56, 57]; renal disease [58]; and respiratory disease [32]) none have applied this across a hospital dietetics department.

To our knowledge, only one study, by Reijnierse et al. (2017), documented barriers to BCA application in practice [43]. These were explored before and after a Dutch health professional training program on detection and management of sarcopenia [43]. Barriers included lack of availability of equipment, lack of knowledge, time constraints, and lack of collaboration with/awareness of other health professionals [43]. When Reijnierse’s study was repeated in a similar sized cohort of Australian and New Zealand health professionals (*n* = 250), as previously found, a lack of diagnostic tools was the main reason for not diagnosing sarcopenia [59]. Lack of sarcopenia awareness and lack of motivation among health-care professionals were also common barriers [43]. In addition to most of these, our study identified additional barriers relating to clinicians’ beliefs about the applicability of the techniques, personal ability to undertake the assessments and confidence in their abilities to incorporate these into their daily practice. Our more extensive suite of barriers may have resulted from a more profession-specific/department-wide assessment rather than training attendees of varied professions [43, 59].

Moreover, applicability issues also relate to BCA validity issues when used with acutely or chronically ill patients. American Society for Parenteral and Enteral Nutrition’s (ASPEN) recent systematic review showed minimal studies that have provided data on BCA in clinical populations. Out of BIA, DXA and ultrasound, DXA and CT scanning were recommended as ‘gold standard’, but the authors indicated that more research is required on the validity of BCA in specific patient populations [36].

Acknowledging the need for addressing all “bottlenecks” (barriers) in each phase of the implementation to ensure diagnosis and management of sarcopenia in daily practices, Reijnierse et al. (2017) highlight the need to draw on the implementation science literature in delivering effective interventions [43]. They highlight that this requires many factors such as acquisition of diagnostic measurement devices, reorganisation of care, collaboration between healthcare professionals, perceived needs and benefits of innovation and organizational factors [43]. Accordingly, we have adopted an implementation science approach to ensure we systematically select interventions that align with identified barriers and enhance existing enablers [48–50].

Following the operationalisation of the evidence-informed strategies to overcome the identified barriers and enablers, our team will progress the overarching interventions of upskilling (professional development strategy), modelling and reducing fear of change (clinical champion project) and embedding as usual practice (departmental integration) the use of BCA to complete a full ‘action cycle’ of the KTA [49, 52]. The details of these strategies are described in Table 1 (column 6). We will repeat our departmental survey in mid-2020 to re-assess adoption of, (perceived) competency in, and attitudes of clinical dietitians towards the utilisation of BCA devices within our department.

A study strength included the use of implementation science methodology and frameworks (KTA, TDF, BCW) [48–50] to map and inform our strategy. Many solutions may appear ‘common sense’ but the systematic assessment and rigour provided by the process provides confidence in the findings and interventions. The survey revealed numerous barriers and enablers to the adoption of BCA in routine clinical care. A greater understanding and/or a wider selection of barriers may have been identified through more qualitative approaches (E.g. focus groups, interviews). However, the methodological approaches were pragmatically chosen to be administered and analysed within routine practice without additional funding. The barriers and enablers identified may reflect specific local departmental issues and may not be generalisable to all sites. However, it is likely that many of these issues are common to other Australian and international sites, as highlighted by Reijnierse [43] and Yeung [59] and colleagues. Study limitations include potential reporting bias or answers reflecting social desirability despite being an anonymous due to the small team size, barriers not existing in the TDF domains allocated to them in the survey, plus lack of data on time burden for dietitians of performing measurements, booking devices, and carrying devices to clinics or wards. We also lack data on objective clinical practice change, resultant clinical outcomes, and cost-effectiveness. Another limitation is the potential impact of knowledge and practice loss with staff turnover; however this was attempted to be circumvented with handover and orientation processes.

## Conclusions

In summary, malnutrition is associated with poorer clinical outcomes in hospitalised patients. BCA devices can be a useful addition to routine clinical care to detect muscle loss that can otherwise be undetected in current malnutrition screening and assessment processes. However, we identified numerous health professional, team, and organisational barriers to the systematic adoption of these processes. Through a process of barrier analysis and intervention mapping within an implementation science framework we have designed three-pronged strategy of dietitian upskilling, embedding and evaluating, and management-endorsement and support to facilitate adoption of practices that will support evidence-based care for these patients. Our next step will be to assess the process of implementation of BCA into routine dietetic practise in our hospital department and its impact on practices, competency, and attitudes of our departmental clinical dietitians with regards to the utilisation of BCA.

## Supplementary Information

**Additional file 1.**

## Data Availability

The datasets used and/or analysed during the current study are available from the corresponding author on reasonable request.

## Abbreviations

BCA: Body Composition Assessment
BCW: Behaviour Change Wheel
BIS: Bioelectrical Impedance Spectroscopy
DXA: Dual Energy X-ray Absorptiometry
FTE: Full Time Equivalents
GLIM: Global Leadership Initiative on Malnutrition
KTA: Knowledge to Action
MUAC: Mid Upper Arm Circumference
MUST: Malnutrition Universal Screening Tool
MST: Malnutrition Screening Tool
PG-SGA: Patient-Generated Subjective Global Assessment
SGA: Subjective Global Assessment
TDF: Theoretical Domains Framework
