# Supplementary material for: Using the theoretical domains framework to inform strategies to support dietitians undertaking body composition assessments in routine clinical care
Source: BMC Health Serv Res. 2021 May 28;21:518. doi: 10.1186/s12913-021-06375-7 (PMC8161923; doi:10.1186/s12913-021-06375-7)
Supplement: Supplementary file 1 — Additional file 1. [file 12913_2021_6375_MOESM1_ESM.docx]

**Appendix A.**

Survey questions on use, attitudes and perceptions on body composition assessment as by dietitians

1. How many years of experience do you have?

- 0-2
- 2-5
- 6-10
- >10

1. What level are you?

- HP3
- HP4
- HP5

1. What area are you working in?

- Babies, Mothers and Women
- Medical/Neurology/Chronic
- Acute/Surgery
- Cancer Care Services
- Management

1. Have you previously had any training in Body Composition and energy assessment?

- Yes
- No

1. If yes, what machines or techniques are these? Please tick all that apply.

- Skinfold thickness
- Arm circumference
- Bio-impedance analysis device (not a scale)
- BIA scale
- DXA
- Other (please specify)

1. Which devices are available to you in the department? Please tick all that apply.

- Skinfold callipers
- Bioelectrical impedance analysis (BIA) device
- Bioelectrical impedance analysis (BIA) scale
- PG-SGA physical exam
- Hang grip strength
- Tape measures

1. How confident are you using these devices when assessing your patients?

|  | Extremely confident | Reasonably confident | Neutral | Not very confident | Not confident at all |
| --- | --- | --- | --- | --- | --- |
| Skinfold callipers | O | O | O | O | O |
| Bioelectrical impedance analysis (BIA) | O | O | O | O | O |
| Mid-upper arm circumference | O | O | O | O | O |
| PGSGA physical exam | O | O | O | O | O |
| Hand grip strength | O | O | O | O | O |
| Other activity measurement device (eg mobile phone or smartwatch) | O | O | O | O | O |
| Tape measures | O | O | O | O | O |

1. How often do you use the following techniques with your patients?

|  | Daily | Monthly | Weekly | Exceptional cases | Never |
| --- | --- | --- | --- | --- | --- |
| Skinfold callipers | O | O | O | O | O |
| Bioelectrical impedance analysis (BIA) | O | O | O | O | O |
| Mid-upper arm circumference | O | O | O | O | O |
| PGSGA physical exam | O | O | O | O | O |
| Hand grip strength | O | O | O | O | O |
| Other activity measurement device (e.g. mobile phone or smartwatch) | O | O | O | O | O |
| Tape measures | O | O | O | O | O |

What stops you from using body composition assessment devices in your day to day practice? Please tick all that apply.

1. Knowledge

- I don't know how to interpret these measurements
- I don't know which patient group I could practice these measurements on
- I don't know when to perform the measurements (i.e. at first assessment only, intervals, frequency)
- I don't know what measurements to perform
- Other (please specify)

1. Skills

- I don't know how to use callipers
- I don't know how to use the hand grip dynamometry
- I don't know how to use a BIA
- I don't know how to use a metabolic cart
- I don't know how to use a tape measure
- I don't know how to use an accelerometer
- Other (please specify)

1. Social/professional role and identity

- The measurements are not required for dietetic assessment
- I think these measures are more appropriate for research
- I do not think these measurements are appropriate for my area of work
- Other (please specify)

1. Beliefs about capabilities

- These measurements are not in my daily routine
- I don't think I could perform these measures accurately
- I think these measurements would be hard for me to learn
- I do not have time to perform these measurements
- Other (please specify)

1. Beliefs about consequences

- I don't think these measurements would benefit my practice/tell me anything new/useful
- We do not have procedures or forms to report these measurements
- Other (please specify)

1. Goals

- I would like to learn more about body composition assessment
- I would like to learn more about energy expenditure assessment
- I would like to apply measurement of body composition and energy expenditure to my practice
- Other (please specify)

1. Memory, attention and decision process

- Body composition assessment does not fit into my schedule (it takes up too much time)
- I forget about doing or scheduling a measurement
- It is too much of a hassle to find a reference value and report the results
- Other (please specify)

1. Environmental context and resources

- I don't have access to the devices I need to perform body composition assessment
- I don't know where these devices are kept
- I don't know how to book these devices
- I know where these devices are kept but I don't know how to get them to the ward
- I don't trust myself not to break the devices
- Other (please specify)

1. Social influences

- My peers do not perform these measurements, so why should I?
- I think these measurements are a burden to patients
- Other (please specify)

1. Intentions

- I never think of doing these measurements when I see or evaluate a patient
- I would like to add these measurements to my daily routine
- Other (please specify)

1. Emotion

- I already have enough on my plate
- I do not need more challenges
- I feel guilty about not performing body composition and energy assessments
- I feel stressed about the time required to do these
- Other (please specify)

1. Optimism

- I do not see the added value of such measurements
- I think it is not feasible to implement these measurements in my practise
- I think these measurements would improve my practise
- I think these measurements would make my practice more interesting
- Other (please specify)

1. Reinforcement

- There's nothing that prompts me to do the measurements
- Having more training available to me would prompt me to do the measurements
- The help of the body comp lab group would make it possible to implement measurements
- Other (please specify)

1. Behavioural regulation

- I'm happy with the way that I assess patient's nutritional status
- I would need to change my practice
- I would need to change my practice regarding assessing nutritional status
- I would need to change my practice regarding assessing energy requirements
- Other (please specify)

1. Do you have any other comments?
2. What would make it easier for you to use body composition assessment devices in your day-to-day clinical practice?
3. How much time do you think it will take to perform these additional assessments?

|  | 1-5mins | 6-10mins | 11-15mins | 15-20mins | 20-25mins | 25-30mins | >30 mins | I’m not sure |
| --- | --- | --- | --- | --- | --- | --- | --- | --- |
| Skinfold callipers | O | O | O | O | O | O | O |  |
| Bioelectrical impedance analysis | O | O | O | O | O | O | O |  |
| Mid-upper arm circumference | O | O | O | O | O | O | O |  |
| PGSGA physical exam | O | O | O | O | O | O | O |  |
| Hand grip strength | O | O | O | O | O | O | O |  |
| Other activity measurement device (e.g. mobile phone or smartwatch) | O | O | O | O | O | O | O |  |
| Tape measures | O | O | O | O | O | O | O |  |

Other comments:

1. How do you think these assessments would change clinical care/patient outcomes?

- Leverage for nasogastric tubes
- Leverage for pre-surgical provision of enteral/parenteral nutrition
- Assist in persuading patients to increase intake/supplements
- Assist in motivation (i.e. to continue on weight loss journey)
- Ability to more accurately assess energy requirements
- Ability to provide objective measures/evaluations of dietetic interventions
- Assist in identifying malnutrition
- I do not expect these measurements to change my practice
- Other (please specify)

1. What patient groups would benefit from using BCE assessments? (Free text)

Skinfold callipers

Bioelectrical impedance analysis (BIA)

Mid-upper arm circumference

PG-SGA physical exam

Hand grip strength

Pedometer

Accelerometer

Total no. minute activity per week

Total no. steps per day

Tape measures

1. Anything else you would like to add? (Free text)

Thank you very much for your input!
